# Screening for several potential pathogens in feral pigeons (*Columba livia*) in Madrid

**DOI:** 10.1186/1751-0147-52-45

**Published:** 2010-06-22

**Authors:** Belén Vázquez, Fernando Esperón, Elena Neves, Juan López, Carlos Ballesteros, María Jesús Muñoz

**Affiliations:** 1 CISA-INIA (Animal Health Research Center). Ctra Algete a El Casar s/n, 28130 Valdeolmos, Madrid, Spain; 2 EQUAM S.L. C/Formentera 1, 28230 Las Rozas, Madrid, Spain

## Abstract

**Background:**

Pathogens with the zoonotic potential to infect humans, such as *Campylobacter jejuni, Campylobacter coli *and *Chlamydophila psittaci*, can be found in feral pigeons (*Columba livia*). Given the high density of these birds in the public parks and gardens of most cities, they may pose a direct threat to public health.

**Methods:**

A total of 118 pigeons were captured in three samplings carried out in 2006-2007 in public parks and gardens in Madrid, Spain. Standard haematological and morphological analyses were carried out on the pigeons. PCR was used to screen for the presence of *Campylobacter jejuni*, *C. coli *and *Chlamydophila psittaci*. Positive samples were confirmed by DNA sequencing.

**Results:**

The analyses demonstrated a high prevalence of *Chlamydophila psittaci *(52.6%) and *Campylobacter jejuni *(69.1%) among the birds captured. In contrast, *Campylobacter coli *was rarely detected (1.1%).

**Conclusions:**

Pigeons in Madrid can carry *Chlamydophila psittaci *and *Campylobacter jejuni*. They may be asymptomatic or subclinical carriers of both pathogens.

## Background

Public parks and gardens are home to abundant populations of birds. One of the most frequent species is the feral pigeon (*Columba livia*), which can be present at densities higher than 2,000 individuals per km^2^, as in Milan [1] or Barcelona [2]. Unfortunately no data are available about pigeon densities in many other major cities, such as Madrid.

Although there are few reports of disease transmission between pigeons and humans [3], their close interaction, together with the observation that these birds are vectors for zoonotic agents [4], may make them a public health risk. In addition, recent work showed that pigeons can cover a maximum distance of 5.29 km [5]; thus, they can spread pathogens locally in their environment.

Thermophilic *Campylobacter *species, especially *Campylobacter jejuni *and *Campylobacter coli*, are considered the primary pathogens responsible for acute diarrhea in the world [6]. In fact, in several countries - e.g. England and Wales, Canada, Australia and New Zealand - *Campylobacter jejuni *infection causes more cases of acute diarrhea annually than do *Salmonella *spp. [7]. In Spain an average of 3,500 cases of *C. jejuni *infection per year has been reported for the period 1989-2001 [7].

In the US, as many as 15% of *Campylobacter *spp. infections may be attributable to contact with companion animals [8]. Reservoirs of *Campylobacter *spp. include a wide range of mammals and birds. However, it remains unclear whether synanthropic birds, in particular feral pigeons (*Columba livia*), act as reservoirs of these pathogens.

*Chlamydophila psittaci *is considered the pathogen most frequently carried by pigeons [9], the "B" serotype the one most often found [9-11]. This serotype has been shown to infect humans; for this reason, non-psittacine birds are thought to be an underestimated source of human chlamydiosis [12].

*Campylobacter jejuni*, *C. coli *and *Chlamydophila psittaci *enter the environment in excrement and, in the case of *C. psittaci*, via ocular and nasal secretions. Transmission to humans can occur by aerosols, direct contact or indirect contact through food and water contamination. To determine the extent to which pigeons might harbour these pathogens and pose a risk to the human population, we screened feral pigeons in Madrid for the presence of several pathogens relevant to public health: *Campylobacter jejuni*, *C. coli *and *Chlamydophila psittaci*.

## Methods

### Birds

Over a 12-month period in 2006-2007, 118 adult feral pigeons of both sexes were captured in several public parks and gardens in Madrid, Spain. The birds were captured in order to evaluate their health status and their potential role as vectors of zoonotic agents.

With permission from the corresponding city council three different samplings using gun-propelled nets [13]were carried out between November 2006 and November of 2007: 62 individuals were captured in November 2006, 27 in May 2007 and 29 in November 2007. All pigeons were ringed for identification in case they were captured again in later samplings.

Pigeons were handled by trained staff, who acted according to standard humane practice designed to minimise stress.

### Sample collection

Blood and cloacal content samples were extracted from each individual. The blood samples were extracted by puncture of the radial vein and were always taken before morphometric measurements were made, in order to prevent changes in haematological parameters due to the stress induced by manipulation of the pigeon. Blood (0.5 ml) was extracted into tubes containing EDTA as anticoagulant. Cloacal samples were obtained by introducing 0.5 ml of sterile DNAse- and RNAse-free phosphate-buffered saline (PBS) into the cloaca, and then retrieving the PBS together with the cloacal contents. The recovered suspension was diluted with PBS to yield a final volume of 2 ml. All samples were transported to the lab under refrigeration.

### Morphological analysis

The following biometric data were recorded for each pigeon: length of the wing cord, length of the tarsus and weight. Two body condition indices were calculated [14,15] (Table 1). The data obtained were compared with the reference data of the Royal Alberta Museum http://www.royalalbertamuseum.ca.

**Table 1:** Morphological results of feral pigeons *(Columba livia) *captured at three different times from public parks in Madrid (n = 118)

	Wing length (mm)	Tarsus length (mm)	Body weight (g)	**BCI^2 ^(g/cm)**^2^	**BCI^3 ^(g/mm)**^3^
This study	221.4 (200-237)	31.6 (27.2-36.3)	275,6 (168-385)	1.24 (0.76-1.86)	8.71 (5.36-11.53)

***Ref***^4^	***228.1 *** ***(225-238)***	***33.1***	***347.9 *** ***(340-356)***	***1.5***^5^	***10.1***^5^

^1 ^Values are shown as means, with ranges given in brackets

^2 ^Body condition index 1 (Weight/Wing length) [14].

^3 ^Body condition index 2 (Weight/Tarsus length) [15].

^4 ^Reference values obtained from http://www.royalalbertamuseum.ca.

^5 ^Calculated as follows: Mean body weight/(Mean wing length or mean tarsus length)

### Haematological analysis

Packed cell volume (PCV) was determined using the hematocrit method at 900 × G for five minutes. Blood was diluted 1:200 with Natt and Herrick solution [16], and red blood cell (RBC) and white blood cell (WBC) counts were made within 24 hours after extraction. Differential WBC counts were made by counting 200 WBCs on Wright-stained smears within the first twelve hours after extraction. Infestation with *Haemoproteus *sp. was estimated by calculating the proportion of affected cells in ten immersion fields (×1000) and multiplying this figure by the RBC count.

### Etiological analysis

Conventional PCR to detect *Chlamydophila psittaci *was carried out as previously described [17], using 200 μl of cloacal enema.

To detect *Campylobacter jejuni *and *C. coli*, 700 μl of cloacal enema were diluted 1:10 in Bolton medium and incubated under microaerophilic conditions for 48 h at 39°C. Then, the bacteria were detected using multiplex PCR as previously described [18].

### Sequencing

PCR products were electrophoresed in 2% agarose gels and stained with SYBR^® ^Green. The specific bands were excised and sequenced. Sequencing was done in triplicate using an ABI Prism 3100 Sequencer (Applied Biosystems). Sequenced products were compared with sequences available in Genbank using BLAST http://blast.ncbi.nlm.nih.gov/Blast.cgi.

### Statistical analysis

Descriptive statistics (minimum, maximum, average, median, and standard deviation) were calculated for the parameters under study. Then chi-square tests were carried out to measure the association between the presence or absence of pathogens and the following independent variables: "sampling", "morphological data" and "haematological data". All statistical studies were conducted using SPSS 15.0 software. When statistically significant differences between seasons were found, correlation tests were carried out separately for each season, since the parameter "season" could be a confounding factor. For all tests, statistical significance was defined as *P *< 0.05.

## Results

Table 1 shows the results of the morphological analysis. Eighty-five point two per cent of the pigeons captured in November 2006, and 100% of the captured in May and November 2007, weighted less than the reference weights of the Royal Alberta Museum. On the other hand, most of the individuals fit within the reference ranges for the various haematological parameters analysed [19] (Table 2). However, the PCV was higher than the upper limit of the normal range in 77% of the pigeons. *Haemoproteus *sp. showed high prevalence (97%). The range of parasitic infestation by *Haemoproteus *sp. was 0-17.2 ×10^5 ^parasites/μl.

**Table 2:** Haematological results of feral pigeons captured at three different times in Madrid.

	Nov-06 (n = 62)	***Reference range***[19]
	*Mean (Range)*	

**PCV (%)**	55.1 (28.6-75.5)	***42-50***
**RBC (×10**^6^**/μl)**	2.9 (1.7-5.4)	***3.1-4.5 ***[34]
**WBC (×10**^3^**/μl)**	11.1 (2.4-47.2)	***9-13***
**Het (%)**	54.1 (20.0-92.0)	***43-63***
**Lym (%)**	43.2 (8.0-78.5)	***42-61***
**Eos (%)**	0.5 (0.0-8.5)	***0-2***
**Bas (%)**	0.9 (0.0-8.0)	***0-2***
**Mon (%)**	1.5 (0.0-8.0)	***0-2***
**Het (×10**^3^**/μl)**	6.3 (0.5-33.3)	***5.9***
**Lym (×10**^3^**/μl)**	4.4 (0.6-13.2)	***5.7***
**Eos (×10**^3^**/μl)**	0.05 (0.0-1.0)	***0.01***
**Bas (×10**^3^**/μl)**	0.08 (0.0-0.4)	***0.0***
**Mon (×10**^3^**/μl)**	0.14 (0.0-1.3)	***0.0***
***Haem *(%)**	5.2 (0.0-95.8)	**-------**
***Haem *(×10**^5^**/ml)**	0.8 (0.0-17.2)	**-------**

Blood samples were tested for the following parameters: packed cell volume (PCV); red blood cell count (RBC); white blood cell count (WBC); percentages and absolute values of different blood cell components, namely heterophils (Het), lymphocytes (Lym), eosinophils (Eos), basophils (Bas) and monocytes (Mon); percentage of erithrocytes infected by *Haemoproteus *spp.; and estimated concentration of *Haemoproteus *spp.

The prevalences obtained for the three pathogens analysed are shown in Table 3. The prevalence of *Campylobacter jejuni *and the mean load of *Haemoproteus *sp. varied significantly across all three sampling times (Statistically significant differences among sampling time for *Haemoproteus *sp.: November 2006- May 2007: p = 0.002; May 2007- November 2007: p = 0.001; November 2006- November 2007: p = 0.001). In contrast, the prevalence of *Chlamydophila psittaci *was similar in November 2006 and November 2007, though it showed a statistically significant decrease in May 2007.

**Table 3:** Prevalence of the three pathogens analyzed in the feral pigeon population in Madrid, sampled at three different times.

Sampling time	*Chlamydophila psittaci*	*Campylobacter jejuni*	*Campylobacter coli*
Nov. 2006	59.7%^a ^(37/62)	81.8%^b ^(36/44)	0% (0/44)
May 2007	37.0%^a ^(10/27)	86.4%^b ^(19/22)	4.5% (1/22)
Nov. 2007	51.8%^a ^(14/27)	35.7%^b ^(10/28)	0% (0/28)

***Overall***	***52.6% (61/116)***	***69.1% (65/94)***	***1.1% (1/94)***

Presence of the pathogens was determined by PCR analysis and sequence alignments with Genbank (see Methods).

^a ^Statistically significant differences among sampling time for *Chlamydophila psittaci*: November 2006- May 2007 (p = 0.030); May 2007- November 2007 (p = 0.049); November (2006-2007)- May 2007 (0.047).

^b ^Statistically significant differences among sampling time for *Campylobacter jejuni*: November 2006- May 2007 (p = 0.032); May 2007- November 2007 (p = 0.001); November 2006- November 2007 (p = 0.001); November (2006-2007)- May 2007 (0.001).

No significant associations between pathogen status and morphological or haematological parameters were found in both separately by month and sampling date and all together analysis.

## Discussion

Animals that live in close contact with humans can be dangerous reservoirs of human pathogens. In this study, we analyzed pigeons (*Columba livia*) captured in urban areas in Madrid to determine the prevalence of three pathogens known to cause disease in humans. Our results show that two of the three pathogens were highly prevalent among the urban pigeons.

The prevalence of *Chlamydophila psittaci *in the three sampling periods is higher than that found in other areas such as Zagreb (15.8%) [20] or Amsterdam (7.9%) [10]. In fact, *Chlamydophila *excretion is intermittent [9], so our results may underestimate the actual prevalence in the pigeon population in our study. At the same time, no significant relationships were observed between *Chlamydophila psittaci *and the morphological or haematological parameters of the pigeons, which may suggest that *C. psittaci *did not affect the health of the pigeons in this study.

The prevalences of *Campylobacter jejuni *obtained in November 2006 and May 2007 in the present study are higher than in previous reports, whereas in November 2007 it was similar to that reported in several locations, including Japan, (23.8-50%) [21], Trinidad (36%) [22] and Barcelona (26.2%) [23]. These values are higher than those reported for Croatia (8.1%) [24], Chile (6.7%) [25] and Oslo (3%) [26]. However, comparison among different areas may be difficult because of the different analytical methods used. The use of PCR following enrichment in Bolton broth, as in the present study, has been shown to be a highly sensitive tool for *Campylobacter jejuni *and *Campylobacter coli *detection [18]. The enrichment may increase the "signal" of viable *Campylobacter *sp. against the "noise" of the biological and chemical complexity of faeces [18]. On the other hand, PCR may amplify dead and uncultivable *Campylobacter *sp., which makes up a certain proportion of stool samples [27].

Our results showed an extremely low prevalence of *Campylobacter coli *for all three sampling times. This echoes previous findings in the literature, such as the study carried out in Barcelona [23], in which no trace of *C. coli *was found.

Analysing *Chlamydophila *sp. prevalence by sampling time indicates a higher prevalence in November. Such variation was reported in a study carried out among pigeons in parks and gardens of Japan, with a prevalence of 100% in November that dropped to 0% by April [28]. This feature may reflect reproductive stress, since *Chlamydophila psittaci *excretion varies as a function of stress level [9], and pigeons can realize up to four egg-layings per year, ending in late autumn (Ballesteros, *pers. com*.).

The lower weight of the pigeons of this study when compared to the Royal Alberta Museum reference data is consistent with the poor condition of the pigeons, and it could also be related to geographical variations in morphological indices for *Columba livia *[29].

Nearly all (97%) of the pigeons studied showed some degree of infestation with *Haemoproteus *sp. The high prevalence of *Haemoproteus *sp. has already been described in the domestic pigeon [30] as well as in other species of wild pigeons [31]. However, the intensity of infestation observed in our study is higher than that previously reported in wild pigeons [31].

In fact, the pathogenic potential of *Haemoproteus *sp. towards its avian hosts has been challenged on numerous occasions. Some authors have not observed any difference in body mass between infected and parasite-free animals [32]. Recent studies suggest that intensity of infection, rather than prevalence, plays a larger role in determining whether the parasite is pathogenic [33]. This previous work, together with the results in the present study, suggest the need for studies involving larger bird populations in order to define the threshold load above which morphological indices may decrease.

The present study demonstrates the extremely high prevalence of two zoonotic pathogens, *Chlamydophila psittaci *and *Campylobacter jejuni*, in feral pigeons in Madrid. At the same time, infection with these pathogens did not appear to be associated with any haematological changes that might reflect immunosuppression, or to any morphological changes that might reflect clinical signs. This leads to the hypothesis that pigeons act as asymptomatic reservoirs of *Chlamydophila psittaci *and *Campylobacter jejuni*. Further studies to estimate the pathogen load are needed, in order to measure pathogen spread in the environment and to estimate the threshold load above which the pigeons may become symptomatic.

## Conclusions

Two zoonotic pathogens are highly prevalent in feral pigeons in Madrid, and the infected pigeons do not show signs of clinical disease. These birds may therefore pose a public health risk to the human population. These data should be taken into account for pigeon population management.

## Competing interests

The authors declare that they have no competing interests.

## Authors' contributions

BV and FE participated equally in the design of the study and helped to write the manuscript. EN coordinated the laboratory work and haematological assays, adapting established PCR techniques in order to detect the pathogens in this study. EN also assisted with the sequence alignments. JL and CB collaborated in the sampling design and helped to write the manuscript. MJM participated in the design and coordination of the overall study and helped to write the manuscript. All authors read and approved the final manuscript.
